# Effects of Sihuang Zhili Granules on the Diarrhea Symptoms, Immunity, and Antioxidant Capacity of Poultry Challenged with Lipopolysaccharide (LPS)

**DOI:** 10.3390/antiox12071372

**Published:** 2023-06-30

**Authors:** Shaolong Wang, Chong Li, Chaosheng Zhang, Guohua Liu, Aijuan Zheng, Kai Qiu, Wenhuan Chang, Zhimin Chen

**Affiliations:** 1 Key Laboratory for Feed Biotechnology of the Ministry of Agriculture and Rural Affairs, Institute of Feed Research, Chinese Academy of Agriculture Sciences, Beijing 100081, China; 82101205294@caas.cn (S.W.); lichong@caas.cn (C.L.); 82101225429@caas.cn (C.Z.); liuguohua@caas.cn (G.L.); zhengaijuan@caas.cn (A.Z.); qiukai@caas.cn (K.Q.); 2 Precision Livestock and Nutrition Laboratory, Teaching and Research Centre (TERRA), Gembloux Agro-Bio Tech, University of Liège, 5030 Gembloux, Belgium

**Keywords:** Sihuang Zhili granule, poultry, oxidative stress, anti-inflammation, antioxidant

## Abstract

A growing interest has been focused on Chinese herbs as alternatives to antimicrobial growth promoters, which are characterized by non-toxic side effects and drug resistance. The purpose of this study was to evaluate the effects of the Sihuang Zhili granule (abbreviated as Sihuang) on diarrhea, immunity, and antioxidation in poultry. Thirty male Leghorn chickens, aged 21 days, were randomly assigned to one of three groups with ten animals each. The control group (CON) received intraperitoneal saline injections, while the LPS-challenged group (LPS) and Sihuang intervention group (SH) received intraperitoneal injections of LPS (0.5 mg/kg of BW) and Sihuang (5 g/kg) at d 31, d 33, d 35, respectively. The control and LPS groups were fed a basal diet, while the SH group was fed a diet supplemented with Sihuang from d 21 to d 35. Analysis of the diarrhea index showed that the addition of Sihuang inhibited the increase in the diarrhea grade and the fecal water content caused by LPS, effectively alleviating poultry diarrhea symptoms. The results of the immune and antioxidant indexes showed that Sihuang significantly reduced the contents of the pro-inflammatory factors TNF- α and IL-1 β, as well as the oxidative stress markers ROS and MDA. Conversely, it increased the contents of the anti-inflammatory factors IL-4 and IL-10, along with the activities of antioxidant enzymes GSH-Px and CAT, thereby enhancing the immune and antioxidant abilities of chickens. Furthermore, Sihuang protected the chicken’s ileum, liver, and immune organs from LPS invasion and maintained their normal development. In conclusion, this study confirmed the antidiarrheal effect of Sihuang in poultry farming and demonstrated its ability to improve poultry immunity and antioxidant capacity by modulating antioxidant enzyme activity and inflammatory cytokine levels.

## 1. Introduction

As a source of high-quality protein, poultry eggs and meat are popular among the public, and contribute greatly to world food security and human nutrition. However, within today’s intensive poultry farming model, animals live in a high-density living environment for a long time, aggravating health problems and oxidative stress in chickens [1]. Studies have shown that oxidative stress is an important cause of many diseases, such as poultry enteritis [2] and liver injury [3]. Oxidative stress leads to decreased antioxidant and immune properties in poultry, which greatly increases the risk of pathogenic bacterial infection, and ultimately affects the health and performance of poultry. Poultry colibacillosis, as one of the major diseases affecting poultry health, has a very high fatality rate [4]. Without proper prevention and treatment, it will bring huge economic losses to the poultry industry. In practice, the prevention and treatment of diseases in poultry farming rely mainly on antibiotics. However, a growing number of countries are banning the use of antibiotic growth promoters because of concerns about the bacterial-drug resistance caused by antibiotics. Chinese patent medicine preparation is a medical product made from herbal extract or herbal medicine, which has the characteristics of safety, non-toxic side effects, and no drug resistance. Studies have shown that Chinese patent medicine preparations have clinical application value in anti-oxidation and anti-inflammation [5,6], and have practical effects on chronic hepatitis [7], pulmonary inflammation [8], and gastrointestinal diseases in humans. Therefore, Chinese patent medicine has great potential as a new antibiotic substitute.

Sihuang is a type of Chinese patent medicine that has the functions of clearing heat and detoxifying, and anti-dysentery properties. Its main components are *Coptis chinensis Franch*, Amur cork tree bark, *Rheum palmatum* L., *Scutellaria baicalensis Georgi*, and *Isatis tinctoria*. Chinese medicine literature records that *Coptis chinensis Franch* [9], Amur cork tree bark [10], *Scutellaria baicalensis Georgi* [11], *Rheum palmatum* L. [12], and *Isatis tinctoria* [13] have the function of clearing heat and detoxifying, which forms the basis of the effect of Sihuang. Although Sihuang was included in the 2020 edition of Chinese Veterinary Pharmacopoeia, there are few studies on their anti-inflammatory and antioxidant effects in poultry breeding. Therefore, the purpose of this study is to study the effect of Sihuang on the anti-inflammatory and antioxidant abilities of poultry, through the stress model of SPF (specific-pathogen-free) chickens induced by endotoxin, so as to provide a data reference for the application and promotion of proprietary Chinese medicine in poultry breeding.

## 2. Materials and Methods

### 2.1. Preparing the Materials

The SPF male Leghorn chickens (21 days old) were purchased from Beijing Boehringer Ingelheim Witon Biotechnology Co., Ltd., Beijing China; the lipopolysaccharide (LPS) was *E. coli* serotype O55:B5, purchased from Sigma-Aldrich Chemical Co., St. Louis, MI, USA; the Sihuang Zhili granules were from Baoding Jizhong Pharmaceutical Co., Ltd., Baoding, China.

### 2.2. Experiment Design and Bird Management

The experiment was conducted at the Nankou test base of the Chinese Academy of Agricultural Sciences, Nankou, China. The animal test methods were formulated by the Feed Research Institute of the Chinese Academy of Agricultural Sciences, Beijing, China. The research report followed the guidelines of ARRIVAL [14].

In the study, a total of 30 male Leghorn chickens, 21 days old and weighing approximately 220 g, were randomly allocated into three groups of ten animals each. The treatments comprised a control group (CON), in which saline was intraperitoneally injected at d 31, d 33, and d 35; an LPS challenge group (LPS), in which LPS (0.5 mg/kg of BW) was intraperitoneally injected at d 31, d 33, and d 35; and finally, a Sihuang intervention group (SH), in which Sihuang dietary (Sihuang at 5 g/kg) was fed from d 21 to d 35, with LPS intraperitoneal injection (0.5 mg/kg of BW) at d 31, d 33, and d 35. Refer to Bai et al. [15] for the use of lipopolysaccharide. According to the product instructions, the appropriate Sihuang granules were evenly mixed into the SPF special diet, as the test diet. The experiment lasted for 14 days, and was fed in a single cage. The control group was fed a special diet for SPF chickens, to meet their nutritional requirements. The nutritional formula of the diet is shown in Supplementary Table S1.

The illumination scheme was controlled at 16 h of illumination and 8 h of darkness until the end of the test. From 21 d to 35 d, the relative humidity was controlled at 50–60%, and the ambient temperature was maintained at 24 ± 2 °C until the end of the test. Waste was cleaned up every day, and the air in the house was kept fresh.

### 2.3. Diarrhea Index

On the 35th day of the experiment, the feces from the dung board under each chicken cage were collected with a self-sealing bag. The feces were dried to a constant weight in the oven at 65 °C, then the fresh weight and air-dried weight of the feces were recorded, and the moisture content of the feces was calculated. Fecal moisture content = (wet weight − air dry weight)/wet weight.

The diarrhea of the chickens was observed and scored by replicate on the 35th day. The diarrhea grade score was as follows: 1 = no diarrhea, normal stool; 2 = mild diarrhea and soft stool; 3 = moderate diarrhea and loose stool, perianal stains; 4 = severe diarrhea and watery stool.

The higher the fecal moisture content, and the higher the diarrhea grade score, the more serious the diarrhea symptoms of the chickens.

### 2.4. Sampling

After fasting for 12 h on the 35th day, the feces of all the treatment groups were collected, to determine fecal moisture. At the same time, samples were collected from chickens in all treatment groups. Blood samples were collected from a vacuum tube (2.5 mL) containing EDTA, and a vacuum tube (5 mL) without anticoagulant, and placed on ice immediately. Serum was collected after centrifugation at 3000 rpm for 10 min in a vacuum tube without anticoagulant, and stored at −20 °C until analysis. After the blood sampling, the broilers were euthanized by electric stunning and immediate manual slaughter, and the liver index, spleen index, thymus index, and bursa of Fabricius were measured, according to the production performance noun terms and metric statistics method for poultry [16]. The immune organ index was calculated by the body weight of the slaughtered fresh immune organs (mg)/the body weight of live chickens (g). Two samples of ileum were collected. One was placed into a frozen tube, frozen in liquid nitrogen, and then stored in a −80 °C refrigerator for analysis of histo-like biochemical indexes. The other was fixed with 10% buffered neutral formaldehyde (pH 7.4), for histomorphological analysis.

### 2.5. Hematological and Serum Biochemical Indexes Analysis

The blood cell components in the anticoagulant blood collected from the EDTA tube were detected using an automatic hematology analyzer (SysmeXT-1800i, Shanghai, China). Alanine aminotransferase (ALT) and aspartate aminotransferase (AST) were measured using the Japanese Olympus AU640 automatic blood biochemical analyzer. We used commercial kits (Shanghai Enzyme Linked Immunobiotechnology Co. Ltd., Shanghai, China) and an automated fluorescence instrument (MultiskanM™ SkyHigh, Thermo Fisher Scientific, Waltham, MA, USA) to detect antioxidant and immune indicators in the preserved serum. Serum markers of oxidative stress included malondialdehyde (MDA), and reactive oxygen species (ROS). Serum antioxidant indicators included glutathione peroxidase (GSH-Px), catalase (CAT), superoxide dismutase (SOD), and total antioxidant capacity (T-AOC). Serum immune indicators included tumor necrosis factor (TNF-α), interleukin-1β (IL-1β), interleukin-4 (IL-4), interleukin-6 (IL-6), and interleukin-10 (IL-10).

### 2.6. Ileal Tissue-Like Immune Indices Analysis

The collected ileal tissue was mixed with the phosphate buffer at a weight (g)/volume (mL) ratio of 1:9, using the German IKA T 10 basic ULTRA-TURRAX^®^ homogenizer at 10,000 rpm (homogenizing time of 10 s, interval of 30 s, repeated 3 times) to prepare the 10% intestinal tissue homogenate. The supernatant of the tissue homogenate was prepared by centrifuge at 4000 rpm for 10 min at 4 °C, using a high-speed refrigerated centrifuge. We used commercial kits (Shanghai Enzyme Linked Immunobiotechnology Co. Ltd.) and an automated fluorescence instrument (MultiskanM™ SkyHigh, Thermo Fisher Scientific, Waltham, MA, USA) to detect inflammatory cytokines in the ileal tissue. The detailed inflammatory cytokine indexes are the same as the serum immune indexes in Section 2.5.

### 2.7. Histopathologic Analysis

The fixed ileal samples were transported to Chengdu Lilai Biotechnology Co., Ltd., Chengdu, China, for further processing. All tissues were dehydrated with increasing concentrations of anhydrous ethanol (75%, 85%, 95%, and 100%), mixed with xylene (AR grade), embedded with paraffin, and prepared into 5 µm thick sections using a rotary microtome (Leica, Germany-2016, Wetzlar, Germany), which were then dewaxed and cleaned. The treated ileal sections were stained with conventional hematoxylin-eosin (H&E), and then the images of the sections were collected using a digital three-lens camera microscope (BA210Digital, MacOdy Industrial Group Co., Ltd., Singapore). The images with ×40 magnification were collected for the analysis and measurement of the areas to be observed. The ileum was measured by two measures, each measuring 10 sets of data, including villus height and crypt depth, and then the villus length/crypt depth ratio (V/C) was calculated.

### 2.8. Statistical Analysis

The data were analyzed using a one-factor ANOVA procedure with the SPSS19.0 software package for Windows (SPSS Inc., Chicago, IL, USA). Significant differences between groups were separated using Duncan’s multiple range test. The differences were considered significant at *p* < 0.05. The graphs were designed using Origin 8.5 (Origin Lab, Berkeley, CA, USA).

## 3. Results

### 3.1. Fecal Index Analysis

The effect of Sihuang on the diarrhea index of chickens is shown in Table 1. Compared with the CON group, LPS stimulation significantly increased the grade of diarrhea and fecal water content in the LPS group (*p* < 0.05); the fecal moisture content in the SH group significantly decreased (*p* < 0.05), while there was no significant difference between the levels of diarrhea (*p* > 0.05). Compared with the LPS group, the diarrhea grade and fecal water content in the SH group were significantly lower (*p* < 0.05).

### 3.2. Histopathologic Analysis

The effect of Sihuang on the ileum morphology of chickens is shown in Table 2. Compared with the CON group, the ileal villus height and V/C value in the LPS group were significantly lower than those in the CON group (*p* < 0.05), and the crypt depth was significantly higher than those in the CON group (*p* < 0.05). There was no significant difference between the SH group and the CON group (*p* > 0.05). Compared with the LPS group, the SH group had significantly higher ileal villus height and V/C value (*p* < 0.05), while the crypt depth was significantly lower (*p* < 0.05). The ileum sections of the three test groups are shown in Supplementary Figure S1.

### 3.3. Blood Routine Test Index Analysis

The effect of Sihuang on the blood routine test of chickens is shown in Table 3. Compared with the CON group, LPS stimulation significantly increased the levels of WBC, GRA, PTL and PCT, and decreased the level of Lym, in the blood of the chickens, while the levels of RBC, WBC, GRA, HCT, PLT, PCT, and Lym decreased significantly in the SH group. Compared with the LPS group, the blood RBC, HGB, and HCT levels in the SH group were significantly increased (*p* < 0.05).

### 3.4. Liver Function Index Analysis

The effect of Sihuang on the liver of chickens is shown in Table 4. Compared with the CON group and the SH group, LPS stimulation significantly increased the contents of ALT and AST in the serum and the liver index of chickens. Compared with the CON group, the serum AST content of the SH group was significantly higher (*p* < 0.05).

### 3.5. Immune Organ Index Analysis

The effect of Sihuang on the immune organs of chickens is shown in Table 5. Compared with the CON group, the spleen index, thymus index, and bursa index of the LPS group were significantly increased (*p* < 0.05), and the spleen index in the SH group was significantly higher than that in the LPS group, but there was no difference in the thymus index and the bursa index between the two groups. Significant difference (*p* > 0.05). Compared with the LPS group, the bursa of Fabricius index of the SH group was significantly lower (*p* < 0.05).

### 3.6. Serum Immune Index Analysis

The effect of Sihuang on the serum cytokines of chickens was shown in Table 6. Compared with the CON and SH groups, the LPS group showed pro-inflammatory factors TNF-α and IL-1β. The levels of IL-6 and IL-6 were significantly increased (*p* < 0.05), while the levels of anti-inflammatory factors IL-4 and IL-10 were significantly reduced (*p* < 0.05). Compared with the CON group, only the anti-inflammatory factor IL-4 was significantly lower in the SH group (*p* < 0.05), while there was no significant difference in the other cytokine indicators between the SH group and the CON group (*p* > 0.05).

### 3.7. Ileum Tissue Immune Index Analysis

The effect of Sihuang on the ileal tissue cytokines of chicken is shown in Table 7. Compared with the CON group, the LPS group showed pro-inflammatory factors TNF-α and IL-1β. The levels of IL-6 and IL-6 were significantly increased (*p* < 0.05), while the levels of anti-inflammatory factors IL-4 and IL-10 were significantly reduced (*p* < 0.05). The SH group only showed pro-inflammatory factor IL-1β. The level significantly increased (*p* < 0.05), while there was no significant difference in other cytokine levels (*p* > 0.05). Compared with the LPS group, the SH group showed pro-inflammatory factors TNF-α and IL-1β. The levels were significantly reduced (*p* < 0.05), while the levels of anti-inflammatory factors IL-4 and IL-10 were significantly increased (*p* < 0.05).

### 3.8. Serum Antioxidant Performance Analysis

The effect of Sihuang on the serum antioxidant performance of chickens is shown in Table 8. Compared with the CON group, LPS stimulation significantly reduced the serum GSH-Px, CAT, SOD, and T-AOC enzyme activities (*p* < 0.05), and significantly increased the content of MDA and ROS (*p* < 0.05) in LPS group chickens. The serum SOD and T-AOC enzyme activities in the SH group were significantly reduced (*p* < 0.05), while there was no significant difference in the GSH-Px, CAT, MDA, or ROS levels between the two groups (*p* > 0.05). Compared with the LPS group, the serum GSH-Px and CAT activities of the SH group chickens were significantly increased, while the serum MDA and ROS contents were significantly reduced (*p* < 0.05).

## 4. Discussion

The results of the diarrhea indicators in this study showed that LPS (Escherichia coli serotype) stimulation led to watery feces in chicken flocks in the LPS group, and the diarrhea grade and fecal water content increased significantly. However, the addition of Sihuang in the chicken diet inhibited the LPS-induced watery diarrhea and increased fecal moisture content, and controlled the grade of diarrhea and fecal moisture content at the same level as the CON group. LPS is the main component of the cell wall of gram-negative bacteria, and has certain antigenic characteristics. In this experiment, E. coli serotype LPS was selected to simulate the stress model of chickens infected with E. coli, and diarrhea was one of the main symptoms of the model. This showed that this experiment successfully established the intestinal stress model induced by LPS in SPF chickens, and verified that Sihuang does have a therapeutic effect on poultry diarrhea. The main components of Sihuang are *Coptis chinensis Franch*, Amur cork tree bark, *Rheum palmatum* L., *Scutellaria baicalensis Georgi* and *Isatis tinctoria*, each of which has unique pharmacological properties. Berberine is an important functional component of the *Coptis chinensis Franch* extract, which has the effects of anti-inflammation, and protecting the liver [17]. Zhang et al. [18] reported that berberine increased the expression of NHE3 and AQP4 in a mouse model of diarrhea, and promoted the absorption of Na^+^ and water in the intestine, thereby exerting an antidiarrheal effect. There are also research reports indicating that *Scutellaria baicalensis Georgi* and rhubarb also have the effect of treating intestinal diseases, such as diarrhea and dysentery.

The intestine is an important digestive organ in animals, and the main site of nutrient absorption in the body, and the functional role of the small intestine is very important. In this study, feeding Sihuang significantly increased the villus height and V/C value of the ileum, significantly reduced the crypt depth of the ileum, and maintained the same villus height and V/C value as those of the CON group. Therefore, the intake of Sihuang by poultry resisted the stimulation of LPS on the ileum, and maintained the normal development of the ileum tissue structure. The intestinal wall of the ileum is composed of a mucosal layer, a submucosa layer, a muscular layer, and a serosa layer, from the inside to the outside. The height of the intestinal villi is positively correlated with the nutrient absorption function of the intestinal segments [19], and the depth of the intestinal crypts is negatively correlated with the cell maturation rate and intestinal secretion function [20]. The intestinal villi height/crypt depth (V/C) value is usually used as an important index to evaluate the degree of intestinal development and digestion and absorption function, and intestinal development and absorption function are positively correlated [21]. Therefore, Sihuang not only stabilizes the tissue structure of the ileum, but also maintains the functionality of the intestinal tract.

The main components of blood include blood cells (red blood cells, white blood cells, platelets) and plasma. The analysis of blood cell components is an indispensable method in veterinary diagnosis. Monitoring blood cell components can effectively evaluate the health status of the body. In this study, blood cell components were analyzed by a blood routine test. The results showed that LPS stimulation significantly increased chicken blood WBC, GRA, PTL, PCT levels, but significantly decreased Lym levels. This may be because the LPS stimulated the activation of the chicken’s humoral immune system, resulting in the enhanced differentiation of various leukocytes in the blood. However, when inflammation occurs, the number of lymphocytes decreases, indicating that the body cannot differentiate enough lymphocytes to participate in the immune response in time, and the immune ability of the body is weakened, and LPS causes damage to the immune system of chickens. Liu et al. [22] reported that the intraperitoneal injection of LPS induced an inflammatory response in laying hens, and the serum levels of pro-inflammatory cytokines in laying hens significantly increased, while blood WBC levels also significantly increased [23]. Zhong et al. [24] also reported similar results: LPS stimulation resulted in a significant increase in blood WBC levels and a significant decrease in Lym levels in rats, and these reports were consistent with our study. The results for the SH group in this study were compared with those of the CON group and LPS group. The blood RBC, HGB, and HCT levels of chickens fed with Sihuang in the SH group increased, which improved the body’s nutrient and oxygen transport capacity, and had a positive effect on the body’s recovery.

Under today’s intensive and industrialized poultry production model, environmental stress will be an important cause of poultry intestinal and liver tissue damage [25,26,27]. The results of this study showed that feeding Sihuang significantly reduced the activities of ALT and AST enzymes in chicken serum, and could stabilize the ALT enzyme activities at the same level as in the CON group. The animal serum ALT and AST enzyme activity levels are important indicators in the diagnosis of liver injury, and any pathological or toxic injury will lead to an increase in ALT and AST activity levels [28]. Therefore, the increase in serum AST and ALT activities in the LPS group in this experiment indicated the liver injury of chickens, but feeding Sihuang maintained the serum ALT and AST activities of chickens at normal levels, and inhibited the damage caused by LPS to chicken livers. It has been reported that berberine has a protective effect on liver injury, and can regulate the concentration of endogenous metabolites in a mouse model of cinnabar-induced liver injury [29]. Zhang et al. [30] also reported that rhubarb significantly reduced the serum ALT and AST activity levels in model mice, and inhibited the infiltration of inflammatory cells, by reducing the number of necrotic cells in the liver of model mice. The results for the liver index in this study showed that LPS stimulation significantly increased the liver index of chickens, and that feeding Sihuang could effectively inhibit the increase in chicken liver index induced by LPS stimulation, and maintain the liver index at the same level as in the CON group. As an antigenic substance, LPS can cause liver damage in animals [31]. Shen et al. [32] found, in the LPS-induced inflammation model test, that pathological changes and tissue swelling appeared in the liver tissue of chickens treated with LPS, which would cause liver weight, and be an important cause of an elevated liver index. Therefore, Sihuang maintains the chicken liver index as normal, which also confirms that Sihuang has the effect of inhibiting LPS in chicken liver damage.

The spleen, thymus, and bursa of Fabricius are important peripheral immune organs in poultry, and the development status of these organs has an important relationship with the immune defense ability of the body [33,34]. In this study, LPS stimulation significantly increased the chicken spleen, thymus, and bursa index, which may be due to the activation of the immune system by the LPS stimulation, and the immune organs’ need to synthesize sufficient antibodies and cytokines. Studies have shown that when the animal body is repeatedly stimulated by LPS, the body’s immune mechanism is activated; the peripheral immune organs represented by the spleen, thymus, and bursa of Fabricius proliferate significantly; the secretion of pro-inflammatory cytokines increases; and inflammation and tissue damage occur [24,35], which is consistent with our findings. However, in this study, the thymus and bursa of Fabricius indexes of chickens fed with Sihuang in the SH group were significantly lower than those in the LPS group, and remained at the same level as in the CON group. This showed that Sihuang resisted the invasion of the body’s immune system stimulated by the LPS, stabilized the immune organ index, and improved the body’s immune system, which was confirmed by the results for the inflammatory cytokines.

The results for the inflammatory cytokines in this study showed that LPS stimulation significantly increased the levels of pro-inflammatory factors (TNF-α, IL-1β, IL-6) in chicken serum and ileum tissue, and significantly decreased the levels of anti-inflammatory factors (IL-4, IL-6, IL-10), because the intraperitoneal injection of LPS activated the immune system of the chicken, causing the body to start an inflammatory response. Studies have shown that LPS stimulation leads to the induction and release of pro-inflammatory factors such as TNF-α, IL-1β, and IL-6 in the intestinal tissue [36,37], causing the liver, kidney, and intestines of animals to appear to have an inflammatory response, which was consistent with our findings. In this study, feeding Sihuang decreased the levels of pro-inflammatory cytokines in the serum and ileum tissue, and increased the level of anti-inflammatory factors, and the inflammatory cytokines TNF-α, IL-1β, and IL-10 were maintained at the same level as in the CON group. Jeong et al. [38] reported that berberine inhibited the phosphorylation of protein kinases such as p38, ERK, and JNK in macrophages by activating protein kinases in macrophages, significantly decreased the expression of pro-inflammatory genes, and inhibited LPS-induced inflammation from occurring; therefore, the anti-inflammatory effect of Sihuang may be realized through the regulation of protein kinase by functional components. Wu et al. [39] pointed out in the report that an appropriate combination of traditional Chinese medicines can enhance each other’s efficacy. Therefore, the anti-inflammatory and antioxidant potential of Sihuang composed of compound prescriptions deserves further study.

In today’s poultry production industry, unbalanced feed nutrition, unsuitable ambient temperature, and high stocking density are the direct reasons for the imbalance in free-radical homeostasis in the body, and oxidative stress [40,41]. In this study, LPS stimulation led to a significant decrease in serum GSH-Px, CAT, SOD, and T-AOC levels, and a significant increase in MDA and ROS levels, in chickens. It indicated that LPS violated the antioxidant defense system of chickens, and induced oxidative stress in the body, which may have an important relationship with diarrhea symptoms in chickens. Studies have confirmed that oxidative stress can damage the immune system and antioxidant system of the body, and can affect the transcription of genes, the conduction of cell signals, the activity of enzymes and biological macromolecules, and the functions of cells and organs. This is an important factor in the occurrence and development of diseases [41,42]. T-AOC reflects the ability of the body’s non-enzymatic antioxidant defense system. SOD, GSH-Px, and CAT, as naturally occurring antioxidant enzymes in the body, can degrade those extremely unstable ROS through their respective enzymatic reaction systems, and then regulate the oxidative and antioxidative balance in the body. MDA is an important product of lipid peroxidation, which can cause cell membrane damage. ROS refers to a kind of one-electron reduction product of oxygen, a general term for oxygen-containing and active substances, and is an important factor of biological oxygen toxicity. MDA and ROS can be used as biomarkers of oxidative stress, to assess the degree of oxidative stress. However, in this study, feeding Sihuang significantly increased the activities of the serum antioxidant enzymes T-AOC and GSH-Px in chickens, significantly decreased the serum levels of the oxidative stress markers ROS and MDA, and reduced the levels of GSH-Px, CAT, ROS, and MDA, maintained at the same level as in the CON group. It showed that Sihuang resisted the oxidative stress response induced by LPS in chickens, and stabilized the antioxidant defense ability of chickens. This may have a significant relationship with several main components of Sihuang. The functional components of Sihuang contain antioxidant active molecules, such as flavonoids and phenols, which regulate oxidative stress-related signals, such as the NF-kB and Nrf2 pathways, to exert antioxidant activity. Zhang et al. [43] reported that berberine can enhance the antioxidant capacity of mice, and alleviate the intestinal injury induced by LPS, by increasing the activity of the GSH-Px enzyme, and reducing the content of MDA in the ileum. It was found that the extracts of *Coptis chinensis Franch* and Amur cork tree bark contain antioxidant components, such as flavonoids and carotenoids [44]. Kwon et al. [45] reported that the combined extract of rhubarb and *Scutellaria baicalensis Georgi* could inhibit the oxidative-stress-related inflammatory response mediated by the NF-kB pathway, and alleviate the symptoms of esophageal mucosal injury in rats, by increasing the activity of antioxidant enzymes, and reducing the content of serum ROS.

## 5. Conclusions

In summarize, the supplementation of Sihuang to the diet effectively alleviated poultry diarrhea symptoms induced by LPS (*Escherichia coli* serotype), This was achieved by increasing the levels of anti-inflammatory factors IL-4 and IL-10, as well as the activities of antioxidant enzymes GSH-Px and CAT. Additionally, Sihuang reduced the levels of proinflammatory cytokines TNF-α, IL-1β, and IL-6, as well as oxidative stress markers ROS and MDA, thus enhancing immune performance and antioxidant capacity. Furthermore, Sihuang exhibited resistance against LPS invasion and damage to the chicken’s ileum intestinal tissue, liver, and immune organs. It maintained the structural integrity of the ileum tissue and showed a certain restorative effect on liver and immune organ damage. Comprised of various Chinese herbal medicines, Sihuang contains abundant functional components. The relationship between its antidiarrheal effect and its anti-inflammatory and antioxidant activities warrants further in-depth investigation.

## Figures and Tables

**Table 1:** The effect of Sihuang on the diarrhea index of chickens (N = 10).

Items	Dietary Treatment			SEM	*p*-Value
	CON	LPS	SH		
Diarrhea grade score	1.33 ^b^	3.33 ^a^	1.17 ^b^	0.262	<0.01
Fecal moisture content	19.66 ^b^	26.73 ^a^	16.00 ^c^	1.695	<0.01

CON = control group; LPS = LPS challenge group; SH = Sihuang intervention group. Different lowercase letters in the same line indicate significant difference (*p* < 0.05), SEM = standard error of means.

**Table 2:** The effect of Sihuang on the ileum morphology of chickens (N = 10).

Items	Dietary Treatment			SEM	*p*-Value
	CON	LPS	SH		
Villus length, μm	854.54 ^a^	717.91 ^b^	862.60 ^a^	24.859	0.003
Crypt depth, μm	128.16 ^b^	150.99 ^a^	112.23 ^b^	6.014	0.004
V/C ratio	6.70 ^a^	4.90 ^b^	7.72 ^a^	0.407	0.001

CON = control group; LPS = LPS challenge group; SH = Sihuang intervention group; V/C = villus length (μm)/crypt depth (μm). Different lowercase letters in the same line indicate significant difference (*p* < 0.05), SEM = standard error of means.

**Table 3:** The effect of Sihuang on the blood routine test of chickens (N = 10).

Items	Dietary Treatment			SEM	*p*-Value
	CON	LPS	SH		
MID, 10^9^/L	15.81	15.21	15.97	0.203	0.314
WBC, 10^9^/L	104.43 ^b^	115.44 ^a^	114.99 ^a^	1.782	0.011
GRA, 10^9^/L	32.43 ^b^	43.26 ^a^	42.20 ^a^	1.850	0.020
PLT, 10^9^/L	12.00 ^b^	24.71 ^a^	27.00 ^a^	2.14	0.004
PCT, L/L	0.01 ^b^	0.02 ^a^	0.03 ^a^	0.011	0.025
LYM, 10^9^/L	60.70 ^a^	56.45 ^b^	56.88 ^b^	0.552	0.001
HGB, g/L	127.33 ^ab^	119.00 ^b^	135.00 ^a^	2.685	0.036
RBC, 10^10^/L	220.33 ^b^	219.29 ^b^	254.00 ^a^	0.057	0.018
HCT, L/L	0.21 ^b^	0.20 ^b^	0.23 ^a^	0.020	0.002

CON = control group; LPS = LPS challenge group; SH = Sihuang intervention group; RBC, red blood cell count; WBC, white blood cell count; HGB, hemoglobin concentration; LYM, lymphocyte; MID, middle cell count; GRA, granulocyte count; HCT, hematocrit; PLT, platelet count; PCT, platelet accumulation. Different lowercase letters in the same line indicate significant difference (*p* < 0.05), SEM = standard error of means.

**Table 4:** The effect of Sihuang on the liver of chickens (N = 10).

Items	Dietary Treatment			SEM	*p*-Value
	CON	LPS	SH		
ALT, U/L	58.72 ^b^	77.02 ^a^	63.04 ^b^	2.388	<0.001
AST, U/L	264.23 ^c^	420.68 ^a^	348.50 ^b^	21.351	<0.001
liver index, %	2.28 ^b^	2.69 ^a^	2.28 ^b^	0.075	0.014

CON = control group; LPS = LPS challenge group; SH = Sihuang intervention group; ALT, alanine aminotransferase; AST, aspartate aminotransferase. Different lowercase letters in the same line indicate significant difference (*p* < 0.05), SEM = standard error of means.

**Table 5:** The effects of Sihuang on the immune organs of chickens (N = 10).

Items, %	Dietary Treatment			SEM	*p*-Value
	CON	LPS	SH		
Spleen index	0.20 ^b^	0.98 ^a^	0.32 ^a^	0.019	0.001
Thymus index	0.34 ^a^	0.48 ^b^	0.80 ^b^	0.038	0.018
Bursa of fabricius index	0.77 ^b^	0.72 ^a^	0.38 ^b^	0.047	0.001

CON = control group; LPS = LPS challenge group; SH = Sihuang intervention group. Different lowercase letters in the same line indicate significant difference (*p* < 0.05), SEM = standard error of means.

**Table 6:** The effects of Sihuang on the serum cytokines of chickens (N = 10).

Items	Dietary Treatment			SEM	*p*-Value
	CON	LPS	SH		
TNF-α, pg/mL	40.13 ^b^	53.94 ^a^	43.96 ^b^	1.965	0.001
IL-1β, pg/mL	374.95 ^b^	520.44 ^a^	428.78 ^b^	17.146	0.001
IL-6, pg/mL	14.53 ^a^	22.36 ^b^	17.19 ^a^	1.014	<0.001
IL-4, pg/mL	139.10 ^a^	86.01 ^c^	106.83 ^b^	6.743	<0.001
IL-10, pg/mL	63.19 ^a^	48.55 ^b^	56.98 ^a^	1.865	<0.001

CON = control group; LPS = LPS challenge group; SH = Sihuang intervention group. Pro-inflammatory factors include TNF-α and tumor necrosis factor; IL-1β, interleukin-1β; and IL-6, interleukin-6. Anti-inflammatory factors include IL-4, interleukin-4; and IL-10, interleukin-10. Different lowercase letters in the same line indicate significant difference (*p* < 0.05), SEM = standard error of means.

**Table 7:** The effects of Sihuang on the ileum tissue cytokines of chickens (N = 10).

Items	Dietary Treatment			SEM	*p*-Value
	CON	LPS	SH		
TNF-α, pg/mL	28.71 ^b^	48.51 ^a^	34.65 ^b^	2.485	<0.001
IL-1β, pg/mL	336.00 ^c^	504.73 ^a^	440.18 ^b^	21.014	<0.001
IL-6, pg/mL	13.16 ^b^	17.71 ^a^	14.16 ^ab^	0.900	0.061
IL-4, pg/mL	113.54 ^a^	80.57 ^b^	114.62 ^a^	5.046	0.001
IL-10, pg/mL	63.46 ^a^	37.95 ^b^	62.81 ^a^	3.15	<0.001

CON = control group; LPS = LPS challenge group; SH = Sihuang intervention group. Pro-inflammatory factors include TNF-α and tumor necrosis factor; IL-1β, interleukin-1β; and IL-6, interleukin-6. Anti-inflammatory factors include IL-4, interleukin-4; IL-10, and interleukin-10. Different lowercase letters in the same line indicate significant difference (*p* < 0.05), SEM = standard error of means.

**Table 8:** The effects of Sihuang on the serum antioxidant performance of chickens (N = 10).

Items	Dietary Treatment			SEM	*p*-Value
	CON	LPS	SH		
GSH-Px, U/mL	121.02 ^a^	83.22 ^b^	106.53 ^a^	4.992	<0.001
CAT, U/mL	65.47 ^a^	41.47 ^b^	58.86 ^a^	2.839	<0.001
SOD, U/mL	355.76 a	245.94 ^b^	269.26 ^b^	14.210	<0.001
T-AOC, mmol Trolox/L	0.10 ^a^	0.06 ^b^	0.07 ^b^	0.005	<0.001
MDA, nmol/mL	6.58 ^b^	8.77 ^a^	7.50 ^b^	0.289	<0.001
ROS	188.04 ^b^	266.90 ^a^	206.17 ^b^	11.053	<0.001

CON = control group; LPS = LPS challenge group; SH = Sihuang intervention group; GSH-Px, glutathione peroxidase; CAT, catalase; SOD, superoxide dismutase; T-AOC, total antioxidant capacity; MDA, malondialdehyde; ROS, reactive oxygen species. Different lowercase letters in the same line indicate significant difference (*p* < 0.05), SEM = standard error of means.

## Data Availability

The original contributions presented in the study are included in the article/Supplementary Material; further inquiries can be directed to the corresponding author.

## Supplementary Materials

The following supporting information can be downloaded at: https://www.mdpi.com/article/10.3390/antiox12071372/s1. Table S1. Analysis composition of basal diets and nutrient level (air-dry basis, %). Figure S1. Morphology and structure of ileum. A = control group; B = LPS challenge group; C = Sihuang intervention group.
